# Investigating and evaluating potential antigen binding sites for monoclonal anti-HER2 antibodies: The LightDock approach

**DOI:** 10.1016/j.csbj.2025.06.001

**Published:** 2025-06-03

**Authors:** Emil Stefańczyk, Agata Mitura, Marta Utratna, Magdalena Staniszewska

**Affiliations:** a SDS Optic, EcoTech Complex, Block A, Głęboka 39, Lublin 20-612, Poland; b Fibiomed, EcoTech Complex, Block A, Głęboka 39, Lublin 20-612, Poland; c Institute of Health Sciences, Faculty of Medicine, The John Paul II Catholic University of Lublin, Konstantynów 1J, Lublin 20-708, Poland

**Keywords:** Molecular docking, *in silico* analysis, Cancer diagnostics, Antigen-antibody interactions

## Abstract

Monoclonal antibodies targeting HER2, a receptor overexpressed in certain cancer cells, have greatly improved the treatment of HER2-positive cancers. In addition, anti-HER2 antibodies play a critical role in diagnostic applications, enabling accurate detection of HER2 expression levels. Advancing antibody-based therapies and diagnostic tools require a thorough understanding of binding interactions, but it remains challenging due to complex antibody protein structure and its flexibility, particularly within their complementarity-determining regions. In this study we utilized LightDock, a molecular docking tool simulating protein-protein interactions which can incorporate flexibility that allows the *in silico* analysis of flexible proteins like antibody. Using LightDock we investigated interaction sites between the recently developed by our group anti-HER2 antibodies and their specific antigen HER2 protein. Despite the high variability in the obtained results, a statistics-based approach identified two recurring HER2 regions as potential binding sites and functionally relevant areas in receptor biology. This variability in predicted docking interfaces reflects the inherent complexity of antibody-antigen interactions. This structure based docking approach provides a cost-effective method to analyze antibody-protein interactions and offers preliminary insight into possible epitopes targeted by the novel anti-HER2 antibodies. However, our data indicates that at this time point further validation using experimental techniques will be beneficial to refine and increase the accuracy of the results obtained *in silico*. This report highlights the value of the computational docking in antibody-protein interaction studies, demonstrating significant potential with present and upcoming advancements in computer-based approaches.

## Graphical Abstract

**Figure:**
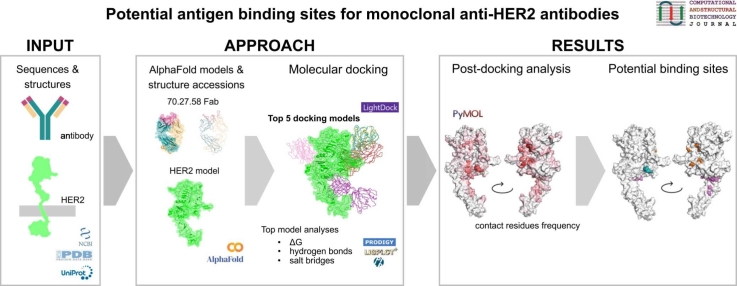

## 1 Introduction

Antibodies play a crucial role in the immune system by recognizing and neutralizing specific antigens associated with intruding factors. The specificity of antibodies, and their high precision in binding molecular target, have led to their use in both therapeutic and diagnostic applications, particularly in oncology. Trastuzumab and Pertuzumab have been utilized as successful antibodies disrupting oncogenic signaling pathways by binding to HER2 (Human Epidermal growth factor Receptor 2) protein overexpressed on cancer cells [11]. Additionally, other monoclonal antibodies are employed in diagnostic methods to measure HER2 levels [34], [4], [41], enabling the stratification of patients and aiding in the personalization of cancer treatments. Both Trastuzumab and Pertuzumab are known to bind to separate regions of the receptor and can be applied simultaneously, as they complement rather than compete in their actions [27], [39]. This feature is critically important, as using Trastuzumab alone may lead to resistance [31], [45]. Understanding the binding mechanism and interaction interfaces of such antibodies is therefore key to refining existing therapies, improving diagnostic accuracy, and enabling the development of novel therapeutic strategies [43].

However, there are significant challenges in determining antibody structural conformations and their specific interaction interfaces due to intrinsic flexibility of these molecules, especially within the complementarity-determining regions (CDRs) which are essential for antigen recognition [21], what leads to multiple possible conformations. Traditional experimental methods, such as X-ray crystallography or cryo-electron microscopy, often yield static snapshots of allowed structures. *In silico* tools for molecular docking generally perform best when applied near-native or holo-like conformations of rigid structures, while performance decreases with apo structures as commonly encountered in antibody modelling. Thus, the challenge lies not only in flexibility but also in the lack of information on the correct binding-competent conformer.

Until 2018, only four full-length IgG antibody structures had been completely determined: PDB entries 1IGT (mouse IgG2), 1HZH (human IgG1), 1IGY (mouse IgG1), and 5DK3 (human IgG4) [19]. Further advancements in techniques have since facilitated the determination of additional full-length IgG structures [46], [6]. The hinge region serves as a flexible linker between the immunoglobulin Fab (fragment antigen-binding) and Fc (fragment crystallizable) regions of the antibody. This flexibility does not constrain their relative positions, enabling the Fab and Fc regions to adopt a variety of spatial configurations [40]. As a result, most experimentally determined antibody structures focus solely on the Fab fragments only.

Computational methods have shown steady progress in modelling protein structures and docking simulations, with antibody-antigen interactions becoming an increasingly active area of research [25]. However, unlike small-molecule drug targets, antibody docking poses unique challenges, such as structural plasticity and the often unknown precise epitope. Many successful docking studies in oncology have focused on small molecules binding well characterised rigid protein pockets, for instance a proteasome inhibitor with a binding mode similar to Bortezomib, as well as selective inhibitors of carbonic anhydrases such as CA IX, which is overexpressed in hypoxic tumours [16], [24], [3], [35], [47].

The computational approach has also been successful in studies of the epidermal growth factor receptor (EGFR/HER1), a tyrosine kinase family member that also includes HER2, HER3, and HER4 [38]. Members of this family can form heterodimers either spontaneously or in response to ligand binding, causing receptor activation leading to downstream signaling pathways involved in cell proliferation and survival. Like other EGFR family members, HER2 protein is composed of an extracellular, membrane, and cytoplasmic domain with tyrosine kinase activity. The extracellular domain (ECD) consists of four subdomains (I–IV, ∼600 amino acids), which play a critical role in receptor dimerization and ligand interactions [44]. EGFR family plays a crucial role in regulating developmental, metabolic, and physiological processes, and protein dysregulations (due to mutations or overexpression) are frequently associated with tumor development. While computational docking has helped elucidate how specific EGFR mutations affect small-molecule inhibitor sensitivity [13], [28], these examples mainly inform drug screening pipelines and do not address the distinct challenges of antibody binding predictions.

In this study, we focus specifically on evaluating the interaction interface between the two anti-HER2 monoclonal antibodies developed by our group [4] and the HER2 extracellular domain (HER2 ECD). For simplicity we refer to HER2 ECD as HER2 throughout the manuscript. The docking simulations were carried out using LightDock [22], a tool that incorporates limited backbone flexibility during simulation, which allows for more realistic modelling of flexible protein-protein interactions than rigid-body-only methods. Our results demonstrate the feasibility and limitations of using such tools to propose interaction interfaces, which can guide downstream experimental validation and refinement. This approach supports antibody-based therapeutic and diagnostic development by contributing to a more targeted understanding of HER2 recognition and monitoring of HER2 expression levels in cancer patients.

## 2 Materials and methods

### 2.1 Input data preparation for antibody and antigen modelling

In this study we have analysed the anti-HER2 antibodies, previously characterized by Antos et al. [4], whose amino acid sequences (Table 1) are deposited in the NCBI GenBank database. The sequences were used to model 3D structures of antibodies using the ColabFold platform providing access to AlphaFold prediction models [29]. For this purpose, the complete sequences of the antibody light chain and partial sequences of the heavy chain (220 of 456 and 218 of 454 amino acids for the 70.27.58 and 70.21.73.67 clones, respectively) were used, with a recycle count of 12, 200 maximum iterations (referring to energy minimization cycles), and the relax option turned on.

**Table 1:** List of the NCBI GenBank accession numbers of the anti-HER2 antibodies examined in this study.

Clone	Chain	Length	Accession number
70.27.58	heavy	456	OR400146.1
	light	218	OR400147.1
70.21.73.67	heavy	454	OR400148.1
	light	214	OR400149.1

The two HER2 protein structures for downstream analyses were downloaded from the RCSB PDB, under the accession numbers 7MN5 [7] and 8Q6J [39]. Both structures were resolved using the electron microscopy at 2.93 and 3.30 Å, respectively. The 7MN5 was originally the HER2 – HER3 heterodimer, out of which HER3 protein was removed in our analysis from the processed structure using PyMOL tool (www.pymol.org). The 8Q6J was on the other hand the HER2 monomer in complex with the Pertuzumab and Trastuzumab, also removed for the purpose of our analysis prior to molecular docking procedures; this structure spanned the 624 aa region of the HER2 protein and covered the complete extracellular fragment of the HER2 protein, encompassing ECD I (annotated by Tashiro et al. at residues 23–198), II (199−340), III (341−508) and IV with missing six C-terminal amino acids (509−652). Neither 8Q6J nor 7MN5 contained the starting N-terminal 22 amino acids and regions spanning transmembrane and cytoplasmic domains. Additionally, the structurally unordered residues were observed in the 7MN5 in the following regions: 121 – 134, 325 – 327, 603 – 612, and following the residue 630.

The third model of HER2 protein structure used was predicted with AlphaFold, using the settings as described above for antibodies. For this purpose, the 1255 amino acids sequence from the UniProt entry P04626 (www.uniprot.org) was retrieved and its 23 – 646 region, as in the 8Q6J structure, was used. The HER2 structure similarities were assessed using PyMOL and its align option that performed a sequence alignment with further structural superposition. The returned measure, root mean square deviation (RMSD) expressed in Angstroms, indicates the average distance between the atoms in queried structures.

### 2.2 Molecular docking

Molecular docking was performed using the LightDock server [22], which supports flexible docking through the Glowworm Swarm Optimization algorithm. This algorithm conducts an exhaustive search within the most promising conformational spaces. The default scoring relied on the DFIRE function, which compares the distance between atoms in a docking model with the distance between the same atom types under ideal conditions. The scoring was biased by the percentage of satisfied residue contact restraints.

The LightDock tool was run using the backbone-flexibility option turned on and with the definition of antibody restraints applied for ligand pre-orientation. The selected restraints corresponded to the CDR amino acids in the 70.27.58 and 70.21.73.67 antibodies, as reported by Antos et al. [4]. The fragments of the 70.27.58 antibody considered the regions of the heavy chain: residues 26 – 33 (8 aa), 51 – 58 (8 aa), and 97 – 109 (13 aa); and the light chain: residues 27 – 36 (10 aa), 54 – 56 (3 aa), and 93 – 101 (9 aa). In case of the 70.21.73.67 antibody, they included the heavy chain: residues 26 – 33 (8 aa), 51 – 58 (8 aa), and 97 – 107 (11 aa); and the light chain: residues 27 – 32 (6 aa), 50 – 52 (3 aa), and 89 – 97 (9 aa). In total, six runs were performed with the two anti-HER2 antibodies and three models of HER2 protein.

### 2.3 Post-docking analysis, visualization and interpretation

The generated docking models were downloaded from the server and the five best from each combination were evaluated using the PRODIGY server that predicts binding affinity in biological complexes, as well as intermolecular contacts in a distance of less than 5.5 Å [18]. In order to identify the hydrogen bonds formed between the receptor and an antibody, the same best models were subjected to LigPlot+ tool [26], while the PLIP server [1] indicated presence of potential salt bridges between interacting partners.

**Fig. 1:**
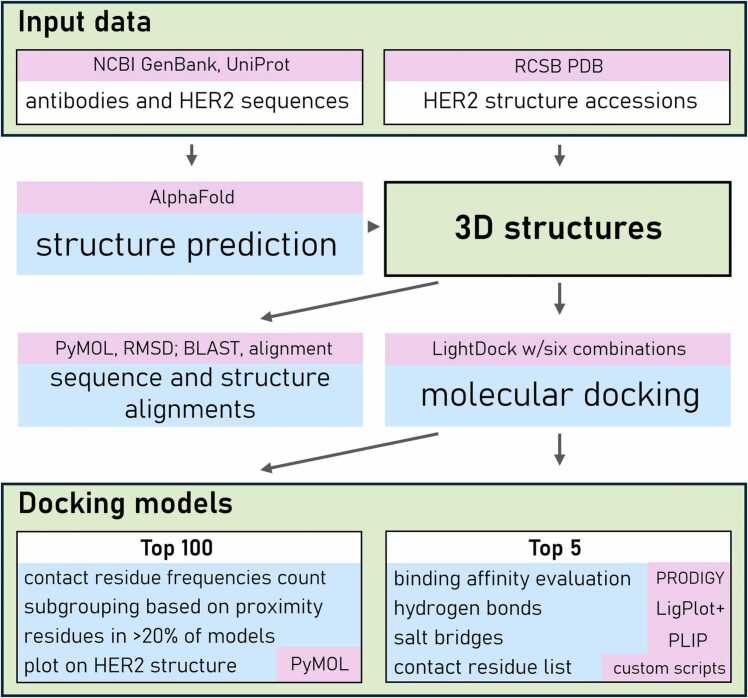
Workflow illustrating the sequential steps of the analysis. Key resources and tools are highlighted using pink background.

To standardize the docking poses generated by the LightDock server, custom in-house scripts were developed using PyMOL. These scripts differed in their approach to presenting results and generating data. The first script facilitated the alignment of docking models after loading them as separate objects. The HER2 protein in the first model served as a reference for aligning the protein structures from subsequent models. This approach enabled the distinct visualization of spatial orientations of differently coloured ligands (residues present within the antibody structure), allowing for visual assessment of whether ligands interacted with the same or neighbouring regions of the HER2 protein, or if alternative binding sites were favoured. The second script focused on identifying the HER2 protein residues positioned within ≤ 2.5 Å of heavy or light antibody chains using the docking models. These residues were highlighted in red, while the HER2 protein was rendered in grey and presented as a transparent mesh for clarity. Ligands (interacting antibodies) were removed to enhance visibility. Additionally, the script generated a list of contact residues, enabling direct comparison across models. These data were further utilized to calculate the frequency of contact residues among the top 100 docking models (the maximum possible in our tool), providing valuable insights into the consistency and preference of antibody–HER2 protein interactions. The Venn diagrams were generated using the online tool available at bioinformatics.psb.ugent.be/webtools/Venn/ and illustrated the interface amino acids shared among models of the same molecular docking combination. All key tools and methodological steps employed in this study are summarized in Fig. 1.

## 3 Results

### 3.1 AlphaFold models of antibodies and HER2 protein

To evaluate the structural properties and accuracy of the antibody models, AlphaFold predictions were analyzed and compared. The relaxed antibody models generated by AlphaFold were assigned high per-residue accuracy (pLDDT) and predicted template modelling (pTM) scores, with values of 95.8 and 0.92 for the 70.27.58 clone, and 95.1 and 0.895 for the 70.21.73.67 clone. Similarly, the HER2 protein model achieved pLDDT and pTM scores of 91.9 and 0.856, respectively.

**Fig. 2:**
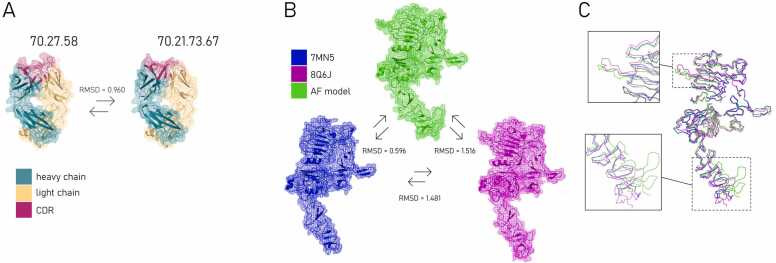
Structural alignment and variability analysis of the *in silico* generated models of anti-HER2 antibodies and HER2 protein structures. (A) Comparison of Fab (Fragment antigen-binding) parts of antibody clones 70.27.58 and 70.21.73.67, showing a root mean square deviation (RMSD) of 0.960 Å. Color-coded regions indicate heavy chain (teal), light chain (yellow), and complementarity-determining regions CDR (magenta). (B) Comparison of HER2 protein extracellular domain (ECD) structures: experimental (7MN5, blue; 8Q6J, magenta) and AlphaFold-predicted model (green), with RMSD values shown for each pairwise alignment. Arrows indicate RMSD values between the two specific HER2 protein structures being compared. (C) Magnified view of variable regions in HER2 protein models, highlighting differences in unstructured residues primarily in 7MN5 (blue).

The models of the antibody clones 70.27.58 and 70.21.73.67 were aligned to each other (Fig. 2A) and to the two therapeutic antibodies, Trastuzumab and Pertuzumab, whose binding positions to the HER2 protein were experimentally validated [39] using structural data derived from the 8Q6J crystal structure shown in Fig. 2B and superimposed in Fig. 2C. All alignment combinations were quantified using RMSD values and are shown in Table 2 (lower triangular part). The upper part of the table presents query coverage and sequence identity results obtained from NCBI BlastP, performed on the concatenated light and heavy chains of each antibody. Basically, the antibody models showed strong consistency with experimentally validated Pertuzumab and Trastuzumab structures, supported by high pLDDT and pTM scores, RMSD values, and sequence identity. The HER2 models also achieved high accuracy scores, indicating structural reliability. These findings suggest the models were suitable for further docking studies.

**Table 2:** The results of the structure (lower triangular part) and sequence (upper – grey cells) alignments expressed in the RMSD and query cover/identity values, respectively.

### 3.2 Molecular docking

**Fig. 3:**
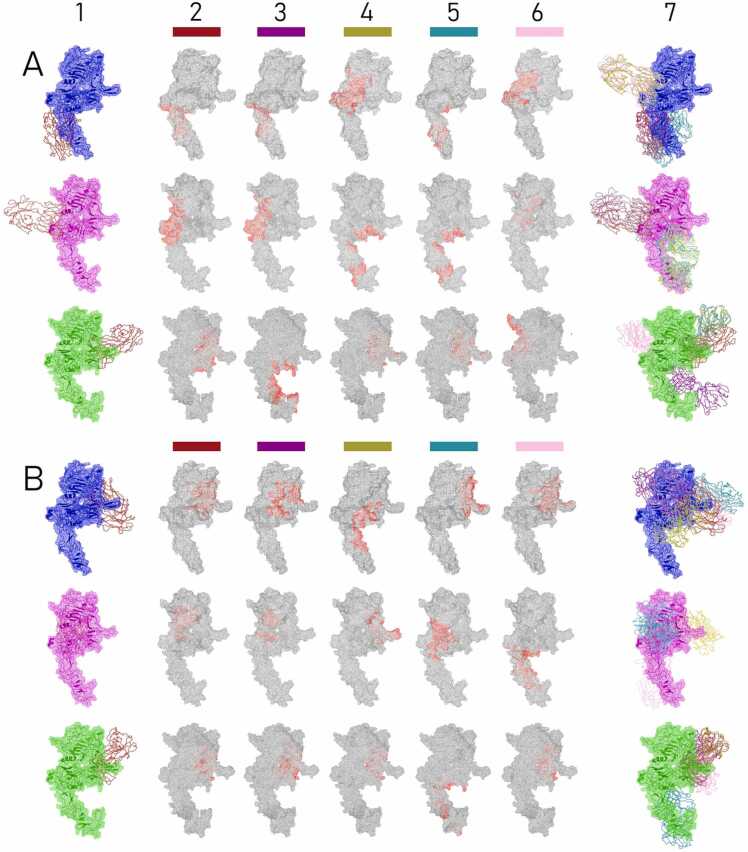
Top molecular docking models of the 70.27.58 (A) and 70.21.73.67 (B) antibodies bound to different models of HER2 structure (three rows in panel A and B). Column 1 depicts positions of the top score docking model of HER2 structure (blue – 7MN5, magenta – 8Q6J or green – AlphaFold model) with the antibody in a ribbon presentation (as indicated by the LightDock score), while column 7 superimposed antibody ribbon structures docking to HER2 protein obtained for the five best models. Columns 2 – 6 represent interface residues (HER2 protein in grey with contact residues in red) individually for five models, shown in LightDock score descending order (from left to right). The colors of each docking model are correlated with their ribbon antibody structure representation shown in the first and last columns.

Altogether, six combinations of a ligand (antibody) and a HER2 protein were run using the LightDock server: two 70.27.58 and 70.21.73.67 antibodies and three HER2 protein structures (7MN5, 8Q6J and AlphaFold model). Complete results were downloaded and analyzed, with the results for top five best models of each combination (in total 30 different combinations) shown in the Table 3 and Fig. 3.

**Table 3:** The summary table containing data from the analysis of five top molecular docking models resulted from molecular docking of the combinations of each antibody clone (70.27.58 and 70.21.73.67) with three models of HER2 protein structures (7MN5, 8Q6J, and AlphaFold) using LightDock server. Following parameters are shown: LD Score – the LightDock server default scoring system, used for ordering the models (1, highest LD Score); ΔG – binding free energy, expressed in kcal/mol; Kd – dissociation constant, expressed in molar at 25°C; IMC – number of intermolecular contacts at the distance below 5.5 Å with specification of contact types, as: Ch (Charged), Pol (Polar), Apol (Apolar). The lower part of the table presents a list of HER2 protein residues (amino acid position in respect to the sequence and a three letter code) in a distance below 2.5 Å from light/heavy chain residues is shown for a given antibody – HER2 protein structure combination model indicated in the first row of the table. The yellow color was used to highlight the residues of the HER2 protein forming hydrogen bonds with an antibody, as indicated by LigPlot+ and their number was counted in the ‘H bonds’ row. The total number of salt bridges in a given model was returned by the PLIP tool [1] and was shown in a corresponding row, while the receptor residues forming such bonds were emphasized with **bold**.

### 3.3 Antibody – HER2 protein binding orientation highlights key interfaces

As shown in Fig. 3, the antibody docking orientation exhibited a diversity of binding positions across models of the same combination and between different HER2 protein structures paired with the same antibody (either 70.27.58, Fig. 3A, or 70.21.73.67, Fig. 3B). Importantly, neither antibody was docked to the C-terminal part of the HER2 protein, which aligns with the exclusion of this region due to its role in anchoring within cell membrane.

**Fig. 4:**
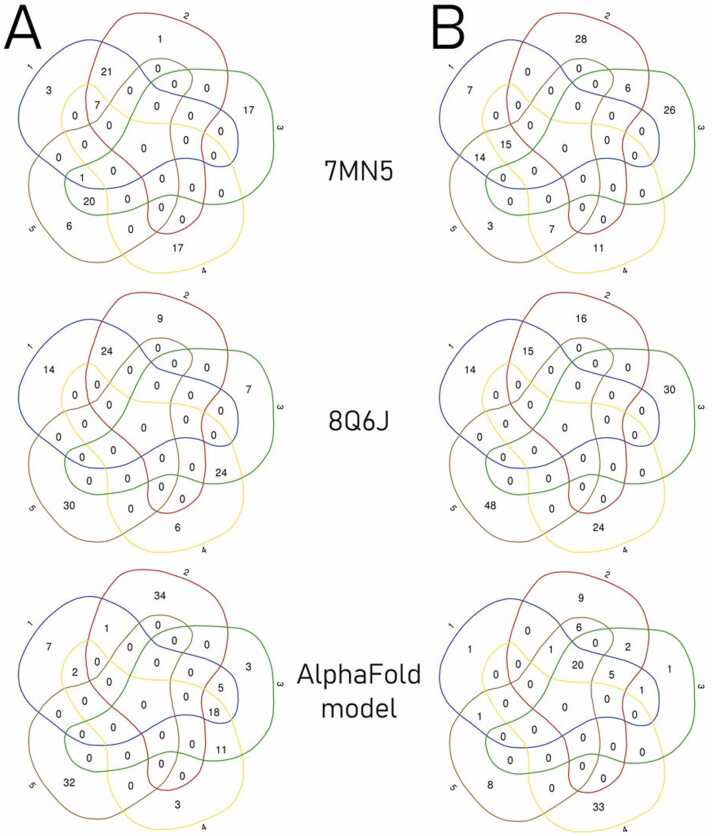
Venn diagrams of residues common for five top models (blue line – highest score model, red – second score model, green – third, yellow – fourth, brown – fifth) resulting from molecular docking of the 70.27.58 (A) and 70.21.73.67 (B) antibodies with the HER2 structures 7MN5 (first row), 8Q6J (second) and AlphaFold-generated model (third), corresponding to the models characterized in Table 3.

In general, a plethora of different interfaces was returned (Fig. 3, column 2–6), and a list of residues involved is shown in section Intermolecular contacts (<2.5 Å) of the Table 3 (based on Supplementary Table 1). Interestingly, the least (24) and the most (48) number of contact residues were identified in models 4 and 5 of the 70.21.73.67 bound to the HER2 8Q6J structure (Fig. 4), with the average of 33 residues counted for models of all combinations. Most of the common residues reported within a single combination was in the 70.21.73.67 docked with the AlphaFold model of the HER2 protein: a total of 20 residues were indicated as forming interface in four of five top models (Fig. 4B, third row). The compatibility was enhanced by additional six residues common between three other models in this combination. Other combinations revealed common residues between at most three models returned, as in the 70.27.58 antibody docked to the 7MN5 (a total of eight common residues), to the AlphaFold model (18), or 70.21.73.67 in combination with 7MN5 (15; Fig. 4).

The number of the hydrogen bonds observed ranged between 1 and 11 with average of 5.9 among all top models (Table 3). Out of a total of 30 docking models, the arginine at position 329 was the amino acid that formed hydrogen bonds in most models (20 %), followed by the threonine at 83 and lysine at 311 (each in 17 %). In turn, the glutamic acid at position 479, histidine at 473 and lysine at 311 were all engaged in forming salt bridges in 13 % of the models.

### 3.4 Binding energy indicates favourable interactions

The ΔG value expressed in kcal/mol describes the change in free energy upon binding between a ligand (here an antibody) and a receptor (here HER2 protein). The more negative values, the stronger the affinity. The negative ΔG values observed across all docking models (reported in the ΔG row of Table 3) suggest energetically favourable binding orientations. ΔG value highly correlated with the number of contact residues within the range of 5.5 Å (r^2^ = 0.84) and within the range of 2.5 Å (r^2^ = 0.66), however it did not reflect the scores returned by the LightDock tool (r^2^ = 0.01). The latter utilized the DFIRE scoring function, a statistical potential that estimates binding energy based on distance-dependent interactions of atom pairs derived from protein structure data, biased by the number of the satisfied residues of docking partners [37], [48].

### 3.5 HER2 protein structure dictates frequent residue involvement in binding

**Fig. 5:**
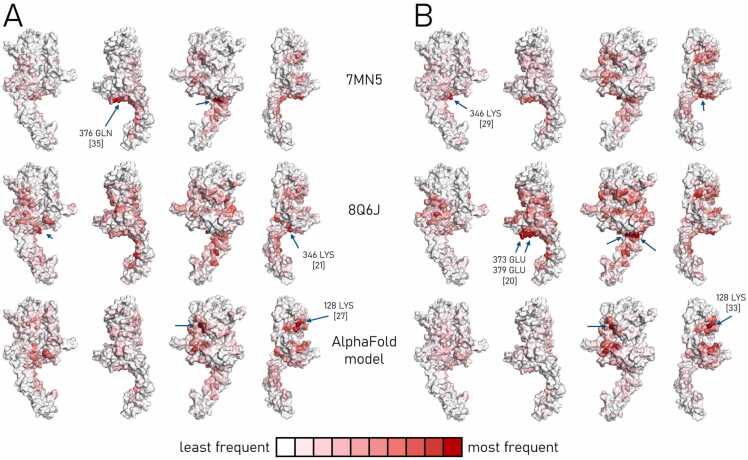
The contact residue frequencies calculated for the top 100 models of each combination of the molecular docking between the 70.27.58 (A) and 70.21.73.67 (B) antibodies and HER2 protein structures 7MN5 (first row), 8Q6J (second) and AlphaFold model (third). Using the intensity of a red color, the frequency of residues involved in formation of the interface was shown, with the position of the most abundant across 100 models indicated with blue arrows and captioned; their number of appearances was presented in the square brackets.

The other approach we have used for identification of the interface relied on counting the most frequent HER2 protein contact residues across 100 models (the maximum number allowed by LightDock) within the same docking combination. The frequencies were then plotted on a HER2 protein structure by using the white-to-red intensity scale with residues appearing most frequently indicated with arrows and labelled (Fig. 5). Interestingly, even though two different antibodies were used for molecular docking with the AlphaFold model of the HER2 protein, the same lysine at position 128 was returned as the most frequent residue (27 appearances per 100 models with the 70.27.58 and 33 with the 70.21.73.67 antibody). The most frequent lysine at the position 346 reported for combination of the 70.21.73.67 antibody with the 7MN5 structure (in 29 models), was also the most frequent in the docking combination between the 70.27.58 and 8Q6J (21 appearances per 100 models). For a better insight into the frequency distributions, a table including the 20 most frequent residues per 100 docking models of each combination was prepared (Table 4), and it included amino acids that were reported in 12–35 docking models. The table revealed that the most frequent residue from one combination, even if not on the first place, quite often appeared within the top 20 residues in other combination, like the most frequent glutamine (position 376 with frequency of 35) in 70.27.58 docked with the 7MN5 structure was reported second-best with frequency of 24, when the same HER2 representation was combined with the 70.21.73.67 antibody.

**Table 4:** Frequency of residues engaged in creating interface between an antibody and one of the HER2 structures counted across the 100 top molecular docking models. Twenty residues per combination in the table were sorted by the number of their appearance and the dashed line demarcated the ones with frequency 20 and above (greyed).

It appeared that the factor of the HER2 protein structure used for molecular docking had stronger impact on the most frequent residues reported from 100 models, than the factor of the antibody. Table 5 presents the number of common residues between different combinations of molecular docking that were previously selected as the most frequent twenty amino acids appearing across 100 top docking models. The highest number of common residues were reported when HER2 protein structure was shared in docking, e.g. the 70.27.58 antibody bound to the 7MN5 receptor shared 12 residues with the same structure, but analysed with the 70.21.73.67 antibody. An exception was the 70.21.73.67-AlphaFold model combination that returned the same number of common residues (9) with the 70.27.58-AlphaFold and 70.21.73.67–7MN5 combinations (Table 5).

**Table 5:** Number of residues, out of 20 most frequent ones across 100 top docking models, shared between molecular docking of various combinations of antibodies and HER2 receptor structure.

		70.27.58			70.21.73.67		
		7MN5	8Q6J	AlphaFold	7MN5	8Q6J	AlphaFold
70.27.58	7MN5		8	5	12	9	6
	8Q6J	8		4	6	10	3
	AlphaFold	5	4		8	5	9
70.21.73.67	7MN5	12	6	8		7	9
	8Q6J	9	10	5	7		4
	AlphaFold	6	3	9	9	4	

### 3.6 Distinct residue groups define potential binding sites

**Fig. 6:**
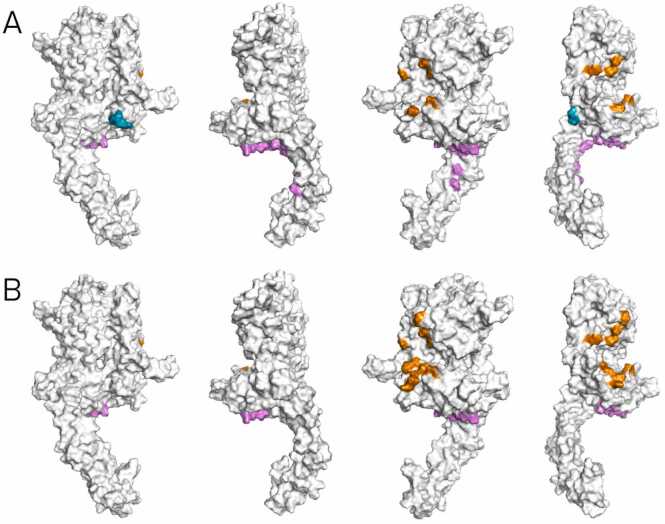
Contact residues shown on the exemplary 8Q6J structure of the HER2 protein observed while docking the 70.27.58 (A) and 70.21.73.67 (B) antibodies to the three HER2 protein structures (7MN5, 8Q6J, AlphaFold), obtained by plotting the 20 (A) and 18 (B) most frequent residues (of frequency above 20 per 100 models). Each colour corresponds to a different residue group that was chosen on basis of their relative position. In each panel there are 4 different planes of the HER2 protein shown for clarity of the antibody contact sites.

Of the most common 20 amino acids of each combination in molecular docking approach, we have selected residues with frequency of 20 and above (Table 4). The residues were grouped according to the antibody used. After removal of the duplicates, 20 and 18 most frequent residues associated with molecular docking of the 70.27.58 and 70.21.73.67 antibodies, respectively, were plotted on the HER2 structure (Fig. 6). By observing the relative proximity, the returned amino acids could be clearly grouped into three distinct residue groups, indicated using orange (group I), violet (group II) and teal (group III) colors. The first two grouped 12 amino acids each (83 T, 128 K, 156 Q, 245 H, 286 V, 288 S, 290 T, 292 V, 294 P, 295 L, 296 H, 329 R and 344 G, 346 K, 373 E, 376 Q, 379 E, 380 T, 404 Q, 504 T, 515 G, 516 Q, 540 P, 543 P), while the third – three amino acids (303 E, 304 D, 305 G). As seen in the Fig. 6, except for the group III residues reported only while docking the 70.27.58 antibody, the most frequent amino acids of the other groups were somehow repeatable, regardless the antibody docked.

## 4 Discussion

### 4.1 Summary of our results

This study used *in silico* docking techniques to investigate the interactions between HER2 and the two novel monoclonal antibodies, originally developed for diagnostic purposes [4], identifying both the advantages and limitations of different computational models. AlphaFold-based structural modelling of two antibody clones (70.27.58 and 70.21.73.67), specifically their Fab (Fragment antigen-binding) parts, and the HER2 extracellular domain (ECD) demonstrated high per-residue accuracy, with pLDDT scores exceeding 91 and pTM scores above 0.85. Structural alignment with experimentally validated therapeutic HER2-binding antibodies (Trastuzumab and Pertuzumab) revealed RMSD values ranging from 0.960 Å (between the two clones) to 1.041 – 4.343 Å (against therapeutic antibodies), suggesting strong structural similarity to Trastuzumab and potential HER2-binding capacity.

In this study, molecular docking simulations using LightDock explored six antibody – HER2 protein combinations, considering three structural representations of HER2 (two crystallographic structures of 7MN5, 8Q6J, and an AlphaFold-predicted model) and Fabs of the two antibodies. Across 30 docking models (top 5 for each combination), binding orientations varied for each antibody, but key interfaces were conserved. The number of intermolecular contacts (2.5 – 5.5 Å) and hydrogen bonds (1 – 11 per complex) were assessed in relation to binding free energy (ΔG), where negative ΔG values confirmed energetically favorable interactions. Contact residue frequency analysis across 100 molecular docking models highlighted recurrent residues at positions 128 (K), 346 (K), and 376 (Q), suggesting structurally preferred binding sites. Based on the distribution of interface residues, the docking results could be grouped into two clusters: one spanning HER2 subdomains I–III, and the other in subdomains III–IV. These loosely resembled known binding patterns of Pertuzumab and Trastuzumab, respectively, but further empirical validation is required [10].

### 4.2 Antibody analysis and observed challenges

A major limitation of this study was the observed variability in docking predictions. While two recurring docking regions on the HER2 protein were identified, our computational analysis identified notable variability in docking outcomes, even when using structurally similar HER2 models. The RMSD range between the three HER2 protein structures was relatively small (0.6 – 1.5 Å); however, the interface surface variations across the top docking models suggested significant differences in predicted interactions. One possible contributing factor was the constraints imposed by the LightDock tool, which required specifying entire CDRs rather than individual binding residues, potentially leading to broader binding predictions [32]. This limitation highlights the need for refined computational approaches that better capture antigen – antibody specificity.

Some additional approaches were taken to study the top five molecular docking models returned for each combination in detail. A correlation was observed between binding energy (∆G) and intermolecular contacts (r² = 0.84 for contacts within 5.5 Å and r² = 0.66 for those within 2.5 Å). While this is in line with the expected behavior of docking scoring functions, it reflects known tendencies to favor models with higher contact numbers rather than necessarily stronger or more specific interactions. However, the number of contacts was not correlated with the number of hydrogen bonds or salt bridges created. Although these types of attractions (salt bridges in particular) are important bonds that can stabilize the connection between docking partners, their number did not appear to correlate with binding energy, which contrasts with prior findings that emphasize the stabilizing role of salt bridges [17]. Similarly, they were not correlated with the score returned by LightDock. Thus, the ΔG calculated with the PRODIGY server was heavily dependent on the number of contacts, but not on the quality of their interactions. As reported by Hendy et al. [17], losing a salt bridge leads to a sharp decline in binding energy; the same authors, however, also observed a decrease in binding energy with a reduction in the number of contacts between an antibody and the receptor binding domain of the SARS-CoV-2 S1 protein. These observations reinforce that docking scores are limited in what they capture and underscore the need for empirical confirmation of computational predictions.

### 4.3 Comparative studies and remaining challenges

The molecular docking results highlight the complexity of antibody – antigen interactions and the challenges inherent in predicting binding site dynamics. While HER2 protein homodimerization has been observed [33], its primary role in oncogenic signaling is through heterodimerization [15], a process that can be inhibited by anti-HER2 antibodies [23]. Furthermore, the distinct binding sites of Pertuzumab and Trastuzumab allow them to be used synergistically to enhance therapeutic efficacy [30], emphasizing the importance of understanding antibody binding mechanisms.

Predicting antibody–antigen binding interfaces remains a significant challenge, particularly due to conformational flexibility and the involvement of residues outside traditional CDRs. Ongoing research is focused on improving the predictive accuracy of docking models. Significant success has been reported for molecular docking of small-molecule ligands that were subsequently validated through wet lab experiments [20], [42], [5], [9]. The study by Ranaudo et al. [36] on affitin-based HER2 mimetics demonstrated the potential of molecular docking and molecular dynamics for predicting binding sites and interaction stability. However, performing docking between larger molecules (like a protein and an antibody) remains very limited in accuracy. This limitation is primarily associated with the flexibility of antibody CDRs, the incidental involvement of residues outside the CDRs in antigen recognition, and the difficulty in receptor epitope prediction due to the multiple possible conformations [2]. Studies have shown that novel tools such as AlphaFold-Multimer, despite significant progress with predicting protein-protein docking, still struggle to accurately model antibody – antigen interactions [8]. The integration of machine learning and deep learning approaches has shown promise in refining antigen – antibody docking simulations [12], [14]. However, these approaches remain at an early stage for antibody modelling and must be supported by experimental validation. While such *in silico* approaches streamline the early-stage characterization of binding partners, they require experimental confirmation using methods such as X-ray crystallography, cryo-electron microscopy (cryo-EM), or nuclear magnetic resonance (NMR).

## 5 Conclusions

This study used molecular docking as a tool to explore possible HER2 binding modes for two novel diagnostic antibodies. The results identified two major binding site groups and highlighted the variability in docking predictions, emphasizing the need for further validation and guiding the experimental design. While trends between contact number and predicted binding energy were observed, their interpretation remains constrained by methodological limitations. While *in silico* approaches accelerate the characterization of antigen – antibody interactions, their reliability depends on integration with experimental methods such as X-ray crystallography, cryo-EM, or NMR. To enhance docking accuracy future research should focus on refining predictive models, particularly through machine learning-enhanced simulations. These advancements will contribute to the development of more precise diagnostic tools and targeted therapies, ultimately improving clinical outcomes in HER2-related cancers.

## Funding

This study was supported by funds from EU, Horizon 2020 SME Instrument grant No. 783818*.*

## CRediT authorship contribution statement

**Marta Utratna:** Writing – review & editing, Writing – original draft, Supervision, Conceptualization. **Agata Mitura:** Writing – review & editing, Conceptualization. **Emil Stefańczyk:** Writing – review & editing, Methodology, Formal analysis. **Magdalena Staniszewska:** Writing – review & editing, Funding acquisition.

## Declaration of Competing Interest

E.S., A.M., M.S. declare financial support from SDS Optic S.A.; A.M., M.S. declare SDS Optic S.A. stock ownership; M.U., M.S. declare financial support from Fibiomed Sp. z o.o.

## Footnotes

Appendix A Supplementary data associated with this article can be found in the online version at doi:10.1016/j.csbj.2025.06.001.
